# Histamine in Fishery: A Five-Year Survey in Northern Italy

**DOI:** 10.3390/toxins16110456

**Published:** 2024-10-25

**Authors:** Sara Morello, Samantha Lupi, Elisa Barcucci, Sandra Fragassi, Elena Torres, Davide Dosio, Chiara Marchese, Tabata Bezzo Llufrio, Marilena Gili, Daniela Manila Bianchi

**Affiliations:** 1 Centro di Referenza Nazionale per la Rilevazione negli Alimenti di Sostanze e Prodotti che Provocano Allergie e Intolleranze—CReNaRiA, Via Bologna 148, 10154 Turin, Italy; samantha.lupi@izsplv.it (S.L.); elisa.barcucci@izsplv.it (E.B.); sandra.fragassi@izsplv.it (S.F.); manila.bianchi@izsplv.it (D.M.B.); 2 Istituto Zooprofilattico Sperimentale Piemonte, Liguria e Valle d’Aosta, Via Bologna 148, 10154 Turin, Italy; elena.torres@izsplv.it (E.T.); davide.dosio@izsplv.it (D.D.); chiara.marchese@izsplv.it (C.M.); tabata.bezzo@izsplv.it (T.B.L.); marilena.gili@izsplv.it (M.G.)

**Keywords:** histamine, foodborne outbreak investigation/foodborne disease, food safety

## Abstract

Histamine is a biogenic amine and an indicator of fishery product freshness and hygienic quality. The European Regulation EC 2073/2005 sets the standards for fish sample collection and establishes quantitative levels of histamine in fishery products to ensure consumer health and safety. This retrospective study presents data on histamine monitoring in fish and fishery products collected in northern Italy between 2018 and 2022. A total of 138 samples were analysed via enzyme-linked immunosorbent assay (ELISA) and then confirmed by high-performance liquid chromatography with diode-array detection (HPLC-DAD). Four samples found positive contained histamine levels above the legal limit. Monitoring via ELISA and HPLC-DAD can efficiently detect histamine in fish and fishery products and protect consumers’ health.

## 1. Introduction

Histamine, 4-(2-aminoethyl)imidazole is a biogenic amine resulting from the enzymatic decarboxylation of L-histidine by a wide range of decomposers (e.g., *Enterobacter aerogenes*, *Klebsiella pneumoniae*) and it is produced in the early stages of tissue degradation. Wrong handling and storage conditions (20–37 °C, pH 4.0–5.5, NaCl) can promote bacterial growth and proliferation and thus increase histamine formation [1,2,3]. Other biogenic amines (e.g., tyramine, putrescine, cadaverine) can also be found in fishbone foods, cheese, meat, fermented products, beer, and wine. Moreover, some fish species (e.g., *Scombridae*, *Engraulidae*, *Clupeidae*) naturally contain high amounts of L-histidine and histamine: this can result in food poisoning after the consumption of fish and fishery products [1,4,5].

In these products, the starting concentration and availability of histamine are related to the bacterial decarboxylase activity on free L-histidine. Histamine is degraded by the enzymatic action of diamine oxidase (DAO) in the human gastrointestinal tract.

High doses of histamine can inhibit the digestive enzyme DAO and therefore the consumption of foods containing high amounts of histamine induces toxic effects similar to an allergic reaction. Symptoms of food pseudo-allergic response associated with histamine poisoning include urticaria, eczema, diarrhoea, and bronchial spasms [6,7].

For instance, scombroid food poisoning is a common foodborne illness following the ingestion of fish from the *Scombridae*, *Scombresocidae*, *Clupeidae*, *Engraulidae*, *Coryfenidae*, and *Pomatomidae* families containing high amounts of histamine. The level of intoxication depends on individual sensitivity and susceptibility, which makes it difficult to determine the histamine concentration that can cause the toxic effect [4,8,9]: 70–1000 mg in one meal may trigger the scombroid poisoning syndrome within minutes to a few hours after ingestion [2,10,11]. Other studies reported that even 8–40 mg can cause slight intoxication, which becomes increasingly severe with higher amounts of histamine ingested [9,12,13,14].

To protect consumer health regulations in force, Regulation EC 2073/2005 and the following amendments (Regulation EC 1441/2007, Regulation EC 1019/2013) established food safety limits for histamine and microbiological criteria of fish and fishery products.

The EC regulation sets a sampling plan of nine units per sample collected over the shelf life of the fresh product and establishes legal limits depending on the category of fish products: 100 mg/kg as the minimum limit (m) and 200 mg/kg as the maximum limit (M) for fishery products from fish naturally containing high amounts of histidine (e.g., *Scombridae*, *Clupeidae*, *Engraulidae*, *Coryfenidae*, *Pomatomidae*, *Scombresosidae*); 200 mg/kg (m) and 400 mg/kg (M) for fishery products that have undergone enzyme maturation in brine and for manufactured products containing the fish of these families [15]. In addition, Regulation EC 1019/2013 specifies criteria for fish sauce produced by fermentation of fishery products: a sampling plan of one unit per sample with a limit of 400 mg/kg, applicable to products placed on the market during their shelf life [16]. In Table 1, an overview of histamine limits and sampling plan established by Regulation EC 2073/2005 and the following amendments are reported.

To assess sample compliance with EC regulations, different safety criteria for histamine are required according to the food category considered. For fishery products from fish species associated with a high amount of histidine, the mean concentration of histamine of the nine portions making the whole sample (*n*), provided by the sampling plan, must be lower than m, set at 100 mg/kg. The number of sample units with a concentration between m and M (*c*) is fixed at 2. None of the samples is allowed to exceed M, set at 200 mg/kg. For fishery products that have undergone enzyme maturation treatment in brine, manufactured from fish species associated with a high amount of histidine, the mean concentration of histamine of the nine portions comprising the whole sample (*n*), as provided by the sampling plan, must be lower than m, set at 200 mg/kg, and the number of sample units with a histamine concentration between m and M (*c*) is fixed at 2. None of the samples is allowed to exceed M, set at 400 mg/kg [15]. In the only sample for fish sauce produced by fermentation of fishery products, the limit is fixed at 400 mg/kg and histamine concentration must be equal to or lower than the limit of 400 mg/kg [16].

As fish and fish products can contain considerable amounts of histamine, its content is among the indicators used to evaluate fishery freshness and hygienic quality according to European food safety norms. The U.S. Food and Drug Administration (FDA) sets more restrictive limits: 50 mg/kg is the defect action level for histamine.

**Figure 1:**
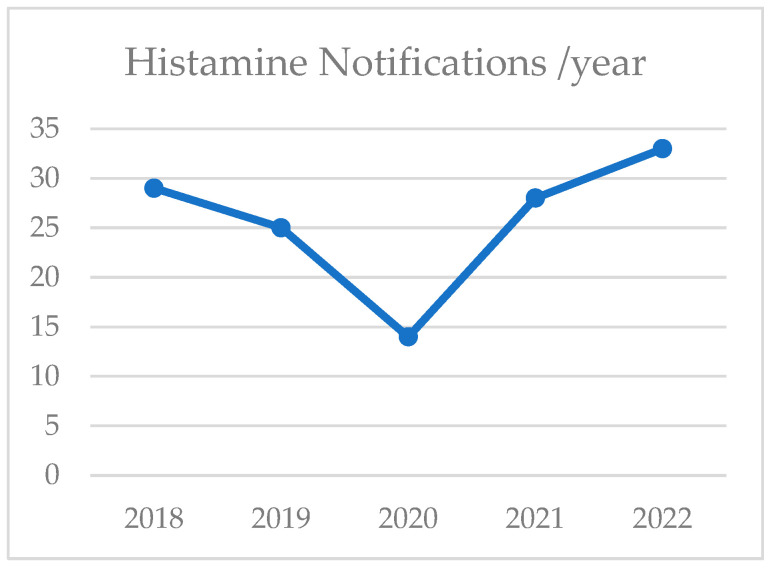
Histamine notifications (no.) between 2018 and 2022 in Europe.

Between 2018 and 2022, the Rapid Alert System for Food and Feed (RASFF) issued an increasing number of histamine notifications in Europe (Figure 1).

Between 2018 and 2022, 129 of 1374 alerts for fish products were due to histamine levels exceeding the maximum limit according to EC regulations. All fish products in the bio-contaminant hazard category had histamine levels above the food safety limits. The mean percentage of histamine notifications during the five-year period was 9.5% of the total notifications concerning fish products.

To evaluate histamine content, different analytical methods can be used to detect histamine levels in foods. Official food safety laboratories often use an immuno-enzyme technique for screening analysis. Histamine concentration is then measured by means of a confirmatory quantitative method, such as HPLC-DAD. The criteria established by Regulation EC 2073/2005 for histamine concentrations (100, 200, 400 mg/kg) refer to HPLC-DAD as the reference method.

This study presents the results of a retrospective survey on histamine monitoring in fish and fishery products between January 2018 and December 2022 in Northern Italy conducted as part of official food safety control [17]. Food samples were screened by enzyme-linked immunosorbent assay (ELISA). Samples found positive for histamine (>50 mg/kg) underwent confirmatory testing with high-performance liquid chromatography with diode-array detection (HPLC-DAD) to identify and quantify histamine concentration and determine compliance with EC regulations.

## 2. Results

### 2.1. Validation of ELISA

The ELISA screening validation method was verified in-house considering the following parameters: specificity, β error at 50 mg/kg concentration level, and ruggedness (Table 2).

Results obtained by analyses to evaluate specificity and β error showed that validation performance criteria were successfully fulfilled, with β error less than 5% according to the decision limit of 50 mg/kg. The kit used is able to discriminate the analyte at our level of interest. Validation results are reported in Table 2. The mean value of B/B0, standard deviation, and RSD% of blank samples and fortified samples are reported in Table 3. The influence of extraction buffer volume, centrifugation speed, and conjugate volume was evaluated in the analysis performed to evaluate method ruggedness, by the Youden approach [18].

### 2.2. Validation of HPLC-DAD

According to Regulation EC 2073/2005, the EN ISO 19343:2017 method is the official method for the determination of histamine [15,19]. However, Article 5 of the same regulation states that “Food business operators may use other sampling and testing procedures, if they can demonstrate to the satisfaction of the competent authority that these procedures provide at least equivalent guarantees”. Therefore, the method internally developed provides a more efficient extraction procedure than the standard method EN ISO 19343:2017 and does not use toluene, which is a toxic substance (IARC).

The confirmatory quantitative HPLC-DAD method was fully validated in-house considering the following parameters: specificity, linearity, precision (repeatability and within-laboratory reproducibility), trueness, and ruggedness (Table 4).

Linearity was evaluated in solvent (perchloric acid 0.4 M aqueous solution) at six concentration levels (0.5–1–5–10–20–40 mg/L) corresponding to matrix concentrations of 5–10–50–100–200–400 mg/kg. Three replicates of the standard calibration curve were considered at each concentration level.

The evaluated parameters, as reported in Table 4, showed that the response was linear in the concentration range (0.5–40 mg/L), with a coefficient of determination of R^2^ = 0.996; the response factor for each concentration level was lower than ±10% of mean Y/X; the limit of detection is lower than the first concentration level of the calibration curve.

The results indicated no interference by the food matrix effect and acceptable specificity. In Table 5, repeatability and recovery analysis data are reported and were considered satisfactory according to internal requirements and parameters. The results of the ruggedness test on eight samples revealed that the slight deliberated variations applied did not have significant changes to method performance and the method is robust. Tests conducted at a concentration level of 50 mg/kg to verify reproducibility showed no significant deviations compared to the standardised method, as reported in Table 6.

Furthermore, upon completion of the validation, the method internally developed has been found to be equivalent to the standardised EN ISO 19343:2017 method.

### 2.3. Results of Samples Analysis

Between 2018 and 2022, a total of 138 fish samples (30 in 2018; 33 in 2020; 18 in 2021; 27 in 2021; 30 in 2022) were collected throughout Northwest Italy and delivered to the Laboratory of the Istituto Zooprofilattico Sperimentale del Piemonte, Liguria and Valle d’Aosta (IZSTO) in Turin. Table 7 reports the number of samples collected during official monitoring in Northwest Italy between 2018 and 2022.

Most (53% of total samples) were collected in the frame of the National Health Services—Regional Monitoring Plan of Food Safety to verify the safety criteria for histamine levels, planned controls (41%), food poisoning/foodborne outbreaks (5%), and border controls (1%). The criterion sampling required by EU regulation of nine samples from each batch was applied. ELISA screening analyses performed on the 138 fish samples showed that 132 (95%) had histamine levels below the CC β of 50 mg/kg and 6 (hereafter ID 1, 2, 3, 4, 5, 6) had levels above the CCβ. Three samples were tested in 2018, two in 2019, and one in 2022. Among the nine units per sample, there were nine in sample ID 2, eight in samples ID 1 and ID 5, six in sample ID 3 and sample ID 6, and one in sample ID 4, for a total of 38 units with histamine content higher than 50 mg/kg.

HPLC-DAD analyses were performed to confirm and quantify histamine in these samples in a quantification range suitable and fit for the purpose of verifying compliance with legal limits established by the EC regulations. According to European Regulations, samples were confirmed compliant when the mean histamine concentration detected is below the m limit, a maximum of *c*/*n* values observed are between the m and M limit, and no values observed exceed the M limit. Table 8 shows the results of HPLC-DAD. In sample ID 1, the mean histamine concentration detected was 1279 ± 180 mg/kg (>m limit; range, 21 ± 4 to 3640 ± 510 mg/kg), and a concentration above the m limit was observed in five out of nine units forming the sample with an amount of histamine exceeding the M limit. In sample ID 2, the mean histamine concentration detected was 838 ± 106 mg/kg (>m limit; range, 234 ± 30 to 1307 ± 183 mg/kg), and a concentration above the m limit was observed in all units forming the sample with an amount of histamine exceeding the M limit. In sample ID 3, the mean histamine concentration detected was 90 ± 12 mg/kg (<m limit; range, 53 ± 7 to 219 ± 29 mg/kg), and a concentration below the m limit was observed in eight units forming the sample, but an amount of histamine exceeding the M limit was observed in one unit. In sample ID 4, the mean histamine concentration detected was lower than the LOQ of the method (20 mg/kg) (LOQ HPLC-DAD) and none of the units forming the sample exceeded the m limit. In sample ID 5, the mean histamine concentration detected was 3802 ± 456 mg/kg (> m limit; range, 964 ± 116 to 7122 ± 855 mg/kg), and a concentration below the m limit was observed in eight units forming the sample with an amount of histamine exceeding the M limit. In sample ID 6, the mean value observed was 74 ± 7.4 mg/kg (<m limit), and a concentration below the m limit was observed in all units forming the sample.

**Figure 2:**
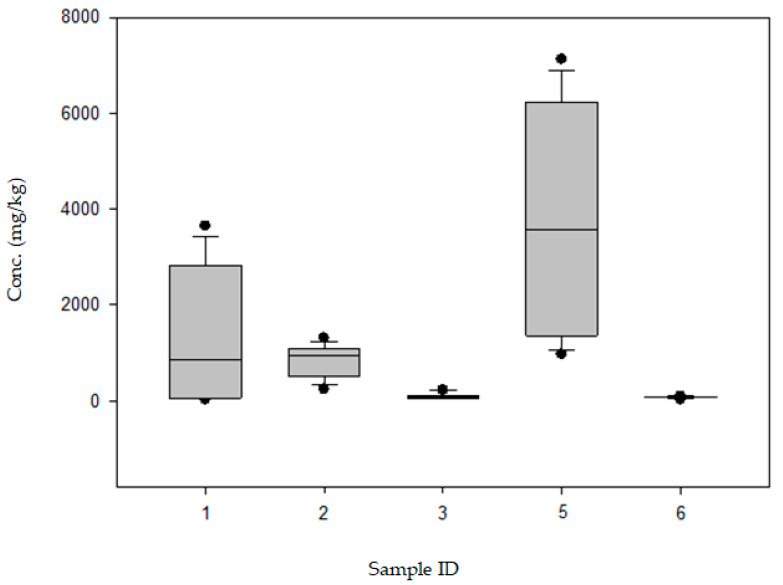
Histamine content (mg/kg) analysed by HPLC-DAD. Sample ID 4 is not reported because the histamine concentration was lower than the LOQ (20 mg/kg) according to HPLC-DAD.

Table 9 presents the statistical data. Statistical analysis was performed using Sigma-Plot software 12.0 (Systat, San Jose, CA, USA). The 9 units per sample were not normally distributed (Shapiro–Wilk, *p* < 0.050). By performing the Kruskal–Wallis test one-way analysis of variance on ranks, we created a boxplot for the clusters (Figure 2). The all pairwise multiple comparison procedure (Dunn’s Method, *p* < 0.05) showed a significant difference between sample ID 5 and samples ID 3 and ID 6. All the other clusters were statistically distant according to this exploration. Nevertheless, the 25th and 75th percentiles were well defined between samples ID 2 (25%, 484 mg/kg) and ID 3 (75%, 125 mg/kg), and ID 6 (75%, 86 mg/kg). In addition, 75% of sample ID 2 (1108 mg/kg) was slightly distant from 25% of sample ID 5 (1293 mg/kg). Samples ID 3 and 6 were characterised by overlapping data. The variance of sample ID 1 (CV% 115) made it difficult to discriminate it from the other samples.

## 3. Discussion

The European Food Safety Authority (EFSA) classifies histamine as a bio-contaminant associated with the toxicological effects of food consumption [20]. Adverse reactions result from the ingestion of food high in histamine content, but acute reactions may manifest in susceptible individuals lacking digestive detoxifying enzymes such as DAO [21,22]. Incorrect food handling and storage conditions can promote the formation of histamine. A major factor in histamine formation is exposure to temperatures that promote microbial growth and proliferation of microorganisms with enzymatic activity.

Histamine formation, directly related to histidine content in fish muscles, occurs postmortem. R Hwang and collaborators reported an increase in histamine after exposure to temperatures (25 °C and 37 °C) and prolonged storage time [13]. Inadequate conditions (temperature, time) of handling and storage can pose a risk to human health and safety [23]. Since histamine is thermostable, heat treatment (e.g., cooking and food processing) does not affect histamine production [24], whereas maintaining the cold chain in food handling and storage can ensure food freshness and quality [25].

For this retrospective study, data were obtained from official monitoring of histamine levels in samples collected between 2018 and 2022. ELISA screening and confirmatory HPLC-DAD proved reliable, effective, and fit for the purpose. Validated analytical methods and accreditation according to ACCREDIA guarantee reliable results.

Most of the samples (95% of the total) were found safe according to the food safety criteria for histamine content. Few samples ( 4, 3% of all samples) contained histamine concentration higher than the m limit set by EC regulations. Non-compliant samples were tuna (*Thunnus albacares*; N = 3) and mackerel (*Scomber scombrus*; N= 1) from Spain: two were analysed during a food poisoning/foodborne outbreak and the two others were sampled as required by Regulation EC 2073/2005. Previous studies reported tuna having the highest histamine content [26]. One sample of tuna fillet analysed in 2018 and one sample of smoked tuna fillet analysed in 2019 were classified as suspected “scombroid poisoning”. The mean histamine levels were 1279 ± 180.3 mg/kg and 3802 ± 456.5 mg/kg, respectively.

The concentration found ranged from 21 ± 3.8 mg/kg to 7122 ± 855 mg/kg and the histamine levels differed considerably among the units per sample because concentrations can vary by parts of a fish sample. In sample ID 1, for example, the lowest histamine concentration was 21 ± 3.8 mg/kg in a unit, while the highest one was 3640 ± 510 mg/kg (mean 1279 ± 180.3 mg/kg) in another unit. In three out of all the units analysed by ELISA, a concentration >50 mg/kg was detected but the correct content was later determined by HPLC-DAD. According to the results obtained, samples ID 1, 2, 3, and 5 were classified as non-compliant and samples ID 4 and 6 as compliant. In sample ID 3, Tukey’s test showed that one unit was an outlier (mean 90 ± 12 mg/kg, range 53 ± 7 to 219 ± 29 mg/kg). Sample ID 5 (2019) showed the maximal concentration of the entire survey.

We observed a wide range in histamine concentration among the units in the non-compliant samples. Histamine formation is related to enzymatic bacterial decarboxylation of L-histidine, which is not equally distributed throughout fish tissue. As a consequence, histamine content in a single sample can vary greatly, and a sampling plan of nine units representing the sample guarantees a more accurate safety assessment. ELISA is a quick and easy method, suitable for screening analyses, giving semiquantitative results. To obtain quantitative information, the HPLC method is needed to confirm the concentration with high sensitivity and accuracy. False ELISA results can be obtained and may be due to interfering factors. Statistical analysis showed that only one sample (ID 5) out of the six found non-compliant by ELISA was significantly different from a sample where all the units were < m (sample ID 4). Therefore, the EU Regulation criteria provide consumer-directed precautional decision making. For example, a very low concentration was found in sample ID 3, except for one unit > m and one > M. The Tukey test showed that the unit > M was not an outlier; nevertheless, sample ID 3 was not statistically different from the compliant samples.

Our findings are shared by observations in previous studies carried out in Italy, in which the incidence of non-compliant samples was the same as that reported by Muscarella and collaborators in a survey conducted between 2009 and 2011 in Puglia: histamine concentration exceeded the legal limit in 11 out of 311 samples or approximately 3.5% of all samples tested [27]. A recent study conducted between 2013 and 2020 in Abruzzo [28] reported that 5.9% of the samples were non-compliant. Other studies reported analogous results, with non-compliant samples accounting for approximately 3% of the total [26]. A similar study carried out in Poland reported histamine content >100 mg/kg in 17.2% of the total samples [29]. An interesting paper investigated histamine content detected in fish samples collected in Southern Italy during the severe acute respiratory syndrome coronavirus 2 (SARSCoV-2) pandemic. The study, conducted on 900 commercially available fish samples collected in 2020, revealed that histamine was detected in 47 fresh tuna samples (5.00%) at levels between 15.07 mg/kg and 596.69 mg/kg. About 1.22% of the samples were over the limits imposed by Regulation (EC) No. 2073/2005 (200 mg/kg for fresh fish products), which is comparable to the multiannual studies conducted in Italy (Cicero et al., 2020b; Lo Magro et al., 2020). High amounts of histamine are due to time and/or temperature abuses during handling and storage (Lo Magro et al., 2020) [30].

## 4. Conclusions

This retrospective survey showed that official monitoring of histamine in fishery products is a useful tool to protect consumer health. Histamine content is an indicator of food quality and freshness. Most of the fish samples collected between 2018 and 2022 were compliant and safe for consumption, whereas the few non-compliant samples had very high histamine levels. Therefore, surveillance and monitoring are central to documenting the compliance of seafood products with EC regulations.

Fully validated methods to test official control samples were used to detect histamine levels in different fish matrices of fishery products, according to food safety criteria (Regulation EC 2073/2005). The ELISA test is suitable for monitoring histamine levels in fish and fishery products and HPLC-DAD provides accurate quantitative results, in compliance with the limits allowed by EC regulations.

Our findings show that the two validated and accredited methods can be effectively combined in official food safety controls, and the quality control system ensures the reliability of results obtained during official controls.

Histamine is a biogenic amine, and it is important to monitor its occurrence in fish products because it can cause intoxication following the consumption of foods containing high levels of this molecule. Suitable validated methods to detect histamine providing reliable results and efficient surveillance procedures are needed to guarantee prompt notification and coordination of competent structures to contain outbreaks.

## 5. Materials and Methods

### 5.1. Sample Collection

A total of 138 fish samples, comprising 68 fresh fish (49.3%) and 70 processed fish (50.7%), were collected between 2018 and 2022. Samples were primarily tuna (*n* = 71, 51%), anchovies (*n* = 17, 12%), pilchard (*n* = 17, 12.3%), mackerel (*n* = 13, 9.4%), and cod (*n* = 6, 4.5%); the remaining 14 samples (10%) included other fish species (herring, swordfish, salmon). Samples are reported in the following Table 10.

They came from several European countries: Spain (53, 38%), Italy (38, 25%), France (8, 6%), Portugal (8, 6%), and Belgium (3, 2%). Approximately 14% of the samples (N = 20) originated from extra-European countries: Morocco (4%, N = 5), Ivory Coast (3%, N = 4), Colombia (2%, N = 3), and Turkey (1%, N = 2). The remaining 4% (N = 5) originated from Tunisia, Mauritius, Peru, Ecuador, and China. Indication of provenance was not available for 6 out of 138 samples. Criterion sampling (nine samples from each batch) was applied according to the guidelines of the EU regulations.

### 5.2. ELISA Screening Analysis

Histamine ELISA kit SENSISpec (Eurofins Technologies, Hungary) is a competitive enzyme immunoassay for the detection of histamine in fresh and processed fish products. All reagents, standards, and plates for the assays are included in the kit. Sample preparation was performed according to the manufacturer’s instructions. Samples positive for histamine (>50 mg/kg) underwent HPLC to quantify histamine concentration in accordance with Regulation EC 2073/2005.

A negative sample was previously tested by HPLC-DAD to verify the absence of histamine and used as the negative quality control. The sample was used to prepare the positive control (C+) at 50 mg/kg of analyte. The negative and the positive quality controls were included in each analytical session and underwent the same analytical procedure described below.

Briefly, samples were homogenised in a mixer (Grindmix GM 200, Retsch Italia Verder Scientific S.r.l., Haan, Germany). Then, 5 g of each sample was transferred into a stomacher bag and 45 mL of deionised water was added. The mixture was shaken at room temperature for 10 min and 1 mL of the extract was then transferred into a 15 mL centrifuge polypropylene tube. After centrifugation at 2000 rcf for 10 min at room temperature, the supernatant (20 µL) was diluted 1:500 with deionised water. Finally, 100 µL of the extracted sample was used for the analysis. In a non-coated plate, standard solutions, samples, and controls underwent derivatisation with an acylation reagent to convert histamine into N-acyl histamine. After incubation for 15 min at room temperature, the derivatised standards, samples, and controls were transferred into a microtiter plate coated with histamine. An anti-histamine antibody was added. Competition for the binding sites between the free *n*-acyl histamine molecules and the histamine coated on the solid phase took 40 min. Unbound molecules were rinsed away. During the second incubation, the antibody antihistamine bound on the solid phase was detected with the addition of an enzyme conjugate. After washing, a chromogenic substrate was added.

Colour development is inversely proportional to the histamine concentration in a sample. Absorbance was measured by a microplate reader (HiPo MPP-96 Microplate Photometer, Biosan, Latvia) at 450 nm within 60 min. The decision limit of the ELISA qualitative method is fixed at 50 mg/kg, corresponding to half of the legal limit of 100 mg/kg. Samples with histamine concentration lower than 50 mg/kg are considered compliant according to EC regulation. Samples with a concentration greater or equal to 50 mg/kg must be subsequently analysed by the HPLC-DAD method to quantify histamine and to verify the compliance limits established by EC regulation. ELISA results are expressed as a percentage ratio between the absorbance measured of sample (B) and the absorbance measured of blank standard (B0). The B/B0 % of samples were compared to the B/B0 % of C+. Samples were considered negative when the B/B0 % value calculated of the sample is higher than the B/B0 % of C+. Samples were considered positive when the B/B0 % value calculated of the sample is lower than the B/B0 % of C+.

### 5.3. HPLC Confirmatory Analysis

To confirm ELISA-positive samples, an HPLC method was used: each sample was analysed with two replicates, and a calibration curve was prepared.

Standard of histamine dihydrochloride (≥95%) was purchased from LGC Standards (Milan, Italy). Acetonitrile, methanol, perchloric acid, sodium bicarbonate, sodium hydroxide, acetone, and dansyl chloride were purchased from Merck (Darmstadt, Germany).

A 1000 mg/L stock solution of histamine was prepared in ultrapure water and stored at 2 ÷ 8 °C for up to 3 months in the dark. Intermediate histamine solution in 0.4 M aqueous perchloric acid was freshly prepared every time by diluting the stock solution to 50 mg/L. Standard working solutions at 2, 5, 10, 20, 40, and 50 mg/L in 0.4 M aqueous perchloric acid were freshly prepared by diluting the intermediate histamine solution.

Dansyl chloride 1% *w*/*v* in acetone was freshly prepared.

For the confirmatory quantitative analysis, 2 g of sample in a 50 mL centrifuge polypropylene tube was homogenised in Ultra Turrax with 20 mL of 0.4 M aqueous perchloric acid, then centrifugated at 3900 rpm for 15 min at room temperature; an aliquot of supernatant was filtered using 0.8 µm syringe filter.

For the derivatisation step, to 1 mL of the filtered sample extract, and to 1 mL of each working standard solution in a 15 mL centrifuge polypropylene tube, 200 µL 2 N aqueous sodium hydroxide, 300 µL sodium bicarbonate solution, and 1000 µL dansyl chloride 1% *w*/*v* in acetone were added, and the mixture was vortexed and incubated at 40 ± 5 °C for 45 min.

After the addition of 150 µL of ammonium hydroxide, the mixture was vortexed and incubated for 1 h at room temperature. Then, 2 mL of acetonitrile was added; the mixture was shaken and centrifugated at 3900 rpm for 10 min. Finally, 1 mL of derivatised extract was transferred into a vial for HPLC-DAD analysis. The extracts were stable up to 72 h when stored at −20 °C.

Analysis was performed with an HPLC chromatograph system (Agilent 1200 series, Agilent Technologies, Santa Clara, CA, USA) with diode-array detection (DAD), using a Luna C-18 (250 × 4.6 mm i.d. 5 µm) column (Phenomenex, Torrance, CA, USA), oven temperature of 25 °C, isocratic elution with water/methanol solution (25/75, *v*/*v*), a flow rate of 1.5 mL/min, injection volume of 30 µL, and DAD wavelength of 254 nm.

### 5.4. ELISA and HPLC-DAD Validation

The ELISA method was fully verified on fishery food matrices according to the EURL Guidance Document on Screening Method Validation Version 1.1, 2023 [31].

The protocol includes the following parameters: method specificity, detection capability (CC β), ruggedness.

Specificity analyses and β error were performed on 22 negative samples, representative of fresh and processed products, including anchovies (N = 3), tuna (N = 3), herring (N = 2), mackerel (N = 2), perch (N = 2), sardine (N = 2), shrimp (N = 2), swordfish (N = 2), bass (N = 1), cod (N = 1), octopus (N = 1), and salmon (N = 1). The same 22 samples were spiked with histamine at 50 mg/kg. Three different analysis sessions were carried out to analyse all samples. The ruggedness of the method was evaluated using the Youden approach [18] and 8 experiments were performed on fishery product samples spiked at 50 mg/kg, applying slight variations on extraction buffer volume, centrifugation speed, and conjugate volume.

The HPLC-DAD method was fully validated as the internal method.

Specificity was determined on 45 samples of fish products; the absence of interferents was verified in the range Δt = Rt ± 2.5%. Linearity was calculated on histamine standard solutions at 6 levels of concentration ranging between 2 mg/L and 50 mg/L (2–5–10–20–40–50 mg/L), corresponding to matrix concentrations between 20 mg/kg and 500 mg/kg. Chromatographic data from the specificity tests were used to determine the limit of detection (LOD); the limit of quantification (LOQ) was calculated by multiplying the LOD by a factor of 3.3.

In order to evaluate precision, accuracy, and recovery, a series of tests on a blank pool of fishery products were conducted at the following fortification levels: LOQ (20 mg/kg), 0.5 times the ML (50 mg/kg), ML (100 mg/kg), 1.5 times the ML (150 mg/kg), 2 times the ML (200 mg/kg), and 4 times the ML (400 mg/kg). Each concentration level was measured on 6 independent replicates in three separate analytical sessions. In each session, the coefficient of variation (CV%) of repeatability for each level was calculated using analysis of variance (ANOVA) [32]; recovery was tested by external standardisation and calculated as the average recovery for each level of concentration.

Ruggedness was evaluated using the Youden approach [18] and 8 experiments were performed on fishery product samples spiked at 50 mg/kg, introducing small changes during sample preparation and analysis. Slight variations (± 10%) were applied on the following seven variables: agitation time (1st extraction step), spinning speed (1st extraction step), derivatisation time, derivatisation temperature, ammonia volume, incubation time, spinning speed (2nd centrifugation).

Finally, the measurement of uncertainty was calculated by a bottom-up method [33] using coverage factors K = 2 and *n* = 2.

## Figures and Tables

**Table 1:** Overview of histamine legal limits (mg/kg) and sampling plan set according to EC regulations. *n* is the number of units comprising the sample and *c* is the number of samples units giving values over m or between m and M.

Food Category	Sampling Plan		Legal Limits (mg/kg)	
	*n*	*c*	m	M
Fishery products from fish species associated with a high amount of histidine	9	2	100	200
Fishery products, except those in food category 1.27a, which have undergone enzyme maturation treatment in brine, manufactured from fish species associated with a high amount of histidine	9	2	200	400
Fish sauce produced by fermentation of fishery products	1	0	400	

**Table 2:** Validation of ELISA performance.

Validation of ELISA Performance	
Specificity	tested on 22 fish samples and fishery
β error	<5%
CC β	<50 mg/kg
Ruggedness	evaluated according to the Youden approach

**Table 3:** Mean value B/B0, standard deviation, and RSD % of blank samples and samples spiked at 50 mg/kg.

Validation Samples	N	Mean Value B/B0	Standard Deviation	RSD %
Blank samples	22	96	5.9	6
Samples spiked at 50 mg/kg	22	51	4.7	9

**Table 4:** Linearity performances and results of the HPLC-DAD quantitative method.

Histamine Concentration mg/L (X)	Analyte Response (Peak Area) (Y)	Response Factor (Y/X)	(Y/X) Mean	$\frac{\left(Y/X\right)-\left(Y/X\;mean\right)\;\%}{\left(Y/X\;mean\right)\;}$	R^2^
0	0	0	46.3	0	0.996
0.5	24	47.7		−2.9	
1	47	46.6		−0.6	
5	232	46.3		0	
10	454	45.4		2.1	
20	916	45.8		1.2	
40	1850	46.2		0.2	

**Table 5:** Validation results of the HPLC-DAD quantitative method.

Concentration Level (mg/kg)	20	50	100	150	200	400
Repeatability limit “r”	2.61	5.52	11.5	20.9	14.9	25.2
Repeatability (RSD%)	3.6	3.0	3.2	3.8	2.0	1.7
Within-laboratory reproducibility (RSD%)	16.5	15.4	4.7	5.8	2.8	7.3
Recovery %	89.9	94.2	93.6	93.1	90.8	92.0
LOD (mg/kg)	6.2					
LOQ (mg/kg)	20.3					
Ruggedness (minor changes)	Method robust					
Linearity	6 concentration levels between 2.0 and 50 mg/l in solvent (corresponding to 20.0–500 mg/kg in matrix): R^2^ ≥ 0.990					
Specificity	Verified on 45 different fish and fishery product samples					

**Table 6:** Validation results of reproducibility.

Conc. Level (mg/kg)	S_R_	Reference S_R_	s_r_	s_r_/σ_r_	Compliance 0.589 ≤ s_r_/σ_r_ ≤ 1.411	Average Accuracy Value %
50	7.688	Primary validation	3.014	0.392	<0.589	95.7%
50	7.74	ISO 19343:2017 method (considering 25 mg/kg tuna, p. 9)	3.014	0.389	<0.589	/

**Table 7:** Sample collection plan in Northwest Italy between 2018 and 2022.

	2018	2019	2020	2021	2022	TOTAL	
Sample Collection Plan	N = 30	N = 33	N = 18	N = 27	N = 30	N = 138	(%)
Regulation EC 2073/2005	13	17	10	15	18	73	53
Veterinary inspection for community compliance	14	14	7	11	11	57	41
Foodborne outbreak	3	2	1	1	-	7	5
Border control	-	-	-	-	1	1	1

**Table 8:** Results of HPLC-DAD analyses and judgment of compliance/non-compliance with EC regulations.

ID Sample	Collection Sample Plan	Type of Fish Sample	Histamine Content (mg/kg)
1	Foodborne outbreak	tuna	58; 48; 21; 3129; 934; 3640; 855; 113; 2713
2	Foodborne outbreak	tuna	1145; 234; 823; 1307; 935; 446; 1060; 522; 1070
3	Regulation EC 2005/2073	mackerel	60; 94; 53; 219; 61; 53
4	Regulation EC 2005/2073	tuna	all units per sample are <20 mg/kg
5	Regulation EC 2005/2073	tuna	5090; 6080; 1490; 6380; 7122; 1227; 2065; 964
6	Regulation EC 2005/2073	herring	82; 91; 27; 86; 62; 86; 86

**Table 9:** Statistical analysis. CV% denotes the coefficient of variation (m = 100 mg/kg, M = 200 mg/kg); 25% and 75% are the 25th and 75th percentiles of statistical distribution.

Sample ID	CV%	Median	25%	75%
1	115	855	53	2921
2	43	935	484	1108
3	72	60,5	53	125
5	69	3578	1293	6305
6	31	86	62	86

**Table 10:** Number of fish samples analysed between 2018 and 2022.

	Year						
Fish	2018	2019	2020	2021	2022	TOTAL	%
Tuna	15	17	12	12	15	71	51
Anchovies	1	6	2	2	6	17	12
Pilchard	3	2	3	6	3	17	12
Mackerel	6	2	1	2	2	13	9
Cod	1	1	-	3	1	6	4
Herring	3	-	-	-	1	4	3
Salmon	1	-	-	-	1	2	1
Swordfish	-	1	-	-	1	2	1
Mullet	-	1	-	-	-	1	1
Sand smelts	-	1	-	-	-	1	1
Prawn	-	-	-	1	-	1	1
Whiting	-	1	-	-	-	1	1
Croaker	-	1	-	-	-	1	1
Surimi	-	-	-	1	-	1	1

## Data Availability

The original contributions presented in the study are included in the article, further inquiries can be directed to the corresponding author.
